# Evolution of Cervical Endoscopic Spine Surgery: Current Progress and Future Directions—A Narrative Review

**DOI:** 10.3390/jcm13072122

**Published:** 2024-04-06

**Authors:** Chuan-Ching Huang, Jamal Fitts, David Huie, Deb A. Bhowmick, Muhammad M. Abd-El-Barr

**Affiliations:** 1 Division of Spine, Department of Neurosurgery, Duke University Hospital, Durham, NC 27710, USA; 2 Department of Orthopedic Surgery, National Taiwan University Hospital, Taipei 100, Taiwan

**Keywords:** cervical, discectomy, decompression, laminoplasty, endoscopic, full endoscopic, percutaneous, minimally invasive spine surgery

## Abstract

Cervical endoscopic spine surgery is rapidly evolving and gaining popularity for the treatment of cervical radiculopathy and myelopathy. This approach significantly reduces muscular damage and blood loss by minimizing soft tissue stripping, leading to less postoperative pain and a faster postoperative recovery. As scientific evidence accumulates, the efficacy and safety of cervical endoscopic spine surgery are continually affirmed. Both anterior and posterior endoscopic approaches have surfaced as viable alternative treatments for various cervical spine pathologies. Newer techniques, such as endoscopic-assisted fusion, the anterior transcorporeal approach, and unilateral laminotomy for bilateral decompression, have been developed to enhance clinical outcomes and broaden surgical indications. Despite its advantages, this approach faces challenges, including a steep learning curve, increased radiation exposure for both surgeons and patients, and a relative limitation in addressing multi-level pathologies. However, the future of cervical endoscopic spine surgery is promising, with potential enhancements in clinical outcomes and safety on the horizon. This progress is fueled by integrating advanced imaging and navigation technologies, applying regional anesthesia for improved and facilitated postoperative recovery, and incorporating cutting-edge technologies, such as augmented reality. With these advancements, cervical endoscopic spine surgery is poised to broaden its scope in treating cervical spine pathologies while maintaining the benefits of minimized tissue damage and rapid recovery.

## 1. Introduction

### 1.1. Cervical Spondylosis

Cervical spondylosis refers to the degeneration of the cervical spine, including the intervertebral disc, facet joint, and spinal ligaments, which may lead to cervical spondylotic radiculopathy and cervical spondylotic myelopathy [1]. Patients with cervical spondylotic radiculopathy typically present with foraminal stenosis, a condition commonly resulting from herniated intervertebral discs at the posterolateral aspect, ucovertebral joint osteophytes, or ligament hypertrophy. The clinical manifestation of this condition includes symptoms such as neck and arm pain and numbness or weakness within the corresponding dermatome or myotome [2]. Alternatively, cervical spondylotic myelopathy, which often stems from spinal canal stenosis due to central herniated discs, osteophyte formation, or ossification of the posterior longitudinal ligament, presents with a more varied symptomatology. These symptoms may range from neck and shoulder pain to loss of hand coordination, gait disturbances, limb motor weakness, and even bowel or bladder dysfunction [3].

### 1.2. Traditional Open Surgery for Cervical Radiculopathy and Myelopathy

Surgical intervention is typically reserved for patients who do not respond to conservative treatment approaches for cervical radiculopathy, those exhibiting progression in their neurological signs and symptoms, or in cases of significant spinal cord compression. Anterior cervical discectomy and fusion (ACDF) has been established as the most common surgical treatment for cervical spondylotic radiculopathy [4]. Posterior cervical foraminotomy has been shown to have comparable clinical outcomes to those of ACDF and is a less invasive procedure with a lower complication profile [5]. Various surgical approaches have been developed to address cervical spondylotic myelopathy. These include discectomy and/or corpectomy and fusion from an anterior approach, as well as laminectomy and fusion or laminoplasty from a posterior approach [6]. Traditional open surgeries have demonstrated favorable clinical outcomes, but they have limitations. For instance, anterior cervical discectomy and fusion inherently increase stress on adjacent segments, potentially leading to adjacent segment disease. The long-term reoperation rate for adjacent segments post-ACDF has been reported at 5.9% over an average follow-up period of 14.5 years [7]. Furthermore, complications associated with the posterior approach often stem from the approach itself, with extensive posterior paraspinal muscle stripping leading to significant postoperative pain and potentially prolonged hospital stays [8,9].

### 1.3. Minimally Invasive Cervical Spine Surgery

Minimally invasive procedures have been introduced to enhance the outcomes of cervical spine surgery. These procedures are designed to minimize trauma to the surrounding tissues while maximizing the surgeon’s ability to achieve the intended surgical objectives. The most frequently performed minimally invasive procedure is the posterior cervical foraminotomy utilizing a tube retractor, a technique introduced by Adamson in 2001 [10]. Studies have demonstrated that the clinical outcomes of this minimally invasive approach are comparable to those of open surgeries [11], with additional benefits such as reduced skin incision size, shorter hospital stays, decreased analgesic use, and less early postoperative neck pain [12]. However, anterior cervical procedures have remained mainly open surgeries due to the proximity of vital structures such as the trachea, esophagus, and vertebral arteries to the cervical spine.

### 1.4. Cervical Endoscopic Spine Surgery

Endoscopic spine surgery has gradually increased in popularity since the 1980s, driven by advancements in optics, surgical instruments, and techniques [13,14,15,16]. The modern era of endoscopic spine surgery began with its application to the lumbar spine. Anthony Yeung introduced the Yeung Endoscopic Spine System (YESS) technique for percutaneous lumbar discectomy in 2003 [17], followed by the advent of the endoscopic interlaminar approach in 2006 [18,19]. Since then, there has been a consistent improvement in both the quality and quantity of endoscopic surgeries performed on the lumbar spine. These endoscopic procedures have been shown to produce clinical outcomes and complication rates comparable to those of traditional open surgeries, with the added benefit of shorter hospital stays in cases involving discectomy [20,21] and decompression for lumbar spinal stenosis [22]. Indications for endoscopic surgery have also expanded to include treatments for infections [23] and tumors [24]. As the surgical techniques for endoscopic procedures in the lumbar spine matured, their application was extended to the cervical spine [25,26]. Both anterior and posterior endoscopic approaches to the cervical spine have been developed [27,28], and novel cervical endoscopic spine surgery techniques have been reported to decrease complications [29], with evidence suggesting promising clinical outcomes [30,31,32]. Cervical endoscopic spine surgery has, thus, established a stable foothold in the realm of cervical spine surgery, with endoscopic procedures for conditions such as cervical disc herniation and intervertebral foraminal stenosis rapidly gaining popularity. Furthermore, incorporating novel imaging systems, navigational aids, and robotic surgeries pushes endoscopic spine surgery to new heights.

Given the rapid evolution in cervical endoscopic spine surgery, this review aims to summarize the various techniques involved, discuss the pros and cons of each, review the current evidence, and explore ways to facilitate these surgeries and enhance patient outcomes.

## 2. Methods

**Figure 1:**
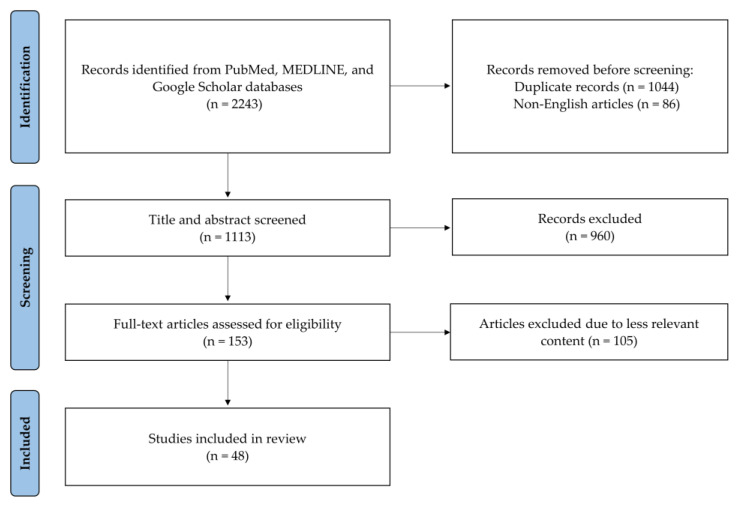
PRISMA flow diagram.

We performed a comprehensive literature search using the PubMed, MEDLINE, and Google Scholar databases in February 2024. No date restrictions were applied, and we included only articles written in English. Our methodology adhered to the PRISMA guidelines for identifying and evaluating relevant studies (Figure 1). The search strategy employed a combination of keywords, including cervical, endoscopy, endoscopic spine surgery, clinical outcomes, complications, limitation, and future. Additional articles were identified by examining the reference lists of articles selected for full-text review. Three researchers (C.-C.H., J.F., and D.H.) initially screened the titles and abstracts to assess articles’ suitability. Subsequently, the full texts of suitable articles were reviewed for inclusion. In instances of disagreement, a fourth researcher (M.M.A.-E.-B.) was consulted to reach a consensus. Finally, 48 articles were included in the review.

## 3. Classification of Endoscopic Spine Surgery

### 3.1. Nomenclature System

Endoscopic spine surgery, as classified within the AO spine nomenclature system, is categorized into three distinct types [33]. The first is the full-endoscopic procedure, which uses an endoscope with an integrated working channel. This channel facilitates the passage of surgical instruments, creating a direct corridor to the surgical site. The remaining types fall under the field of endoscope-assisted surgery, wherein surgical tools are maneuvered through trajectories separate from the endoscope. This category includes microendoscopic procedures, where an endoscope is attached to a tubular retractor, and biportal endoscopic surgery, which utilizes separate viewing and working portals for the arthroscope and surgical instruments. This review will primarily focus on full-endoscopic procedures.

### 3.2. Advantages of Full-Endoscopic Cervical Spine Surgery

Full-endoscopic spine surgery is a minimally invasive technique compared to traditional spine surgeries, as it requires only a small incision. This approach significantly reduces the need for extensive soft tissue stripping, thereby minimizing muscular damage, blood loss, and scarring [34,35]. Such reductions contribute to a decrease in postoperative axial neck pain and an enhancement in postoperative recovery. Compared to conventional open surgery, endoscopic spine surgery is associated with a shorter hospital stay and facilitates an earlier return to work [36,37]. The utilization of continuous intraoperative irrigation not only helps control bleeding through hydrostatic pressure but also, with the use of continuous saline irrigation, significantly reduces the risk of postoperative infection [38]. The proximity of the endoscope to the target site provides surgeons with an intricate view of anatomical features such as epidural fat, small vessels, and the thecal sac. This enhanced visibility aids in delicate hemostasis and helps prevent damage to neural elements. As the advantages of full-endoscopic cervical spine surgery become increasingly recognized, its popularity continues to grow. Adaptations of both anterior and posterior endoscopic techniques are being utilized to address various cervical spine pathologies, reflecting the evolving landscape of spinal surgical practices.

## 4. Common Endoscopic Techniques in the Cervical Spine

### 4.1. Posterior Endoscopic Cervical Foraminotomy/Discectomy (PECF/PECD)

While anterior cervical discectomy and fusion (ACDF) has long been the standard treatment for single-level unilateral cervical radiculopathy [39], posterior foraminotomy has also shown its strength in this domain. A meta-analysis conducted by Sahai et al. [40] revealed that minimally invasive posterior cervical foraminotomy resulted in significantly greater improvements in the visual analog scale (VAS) in arm pain than ACDF did while maintaining similar improvements in VAS-neck and neck disability index (NDI) scores. Another meta-analysis by Fang et al. [41] underscored the advantages of posterior cervical foraminotomy, such as shorter operation time and reduced length of hospital stay, in addition to comparable VAS and NDI improvements and a similar complication rate to that of ACDF.

**Figure 2:**
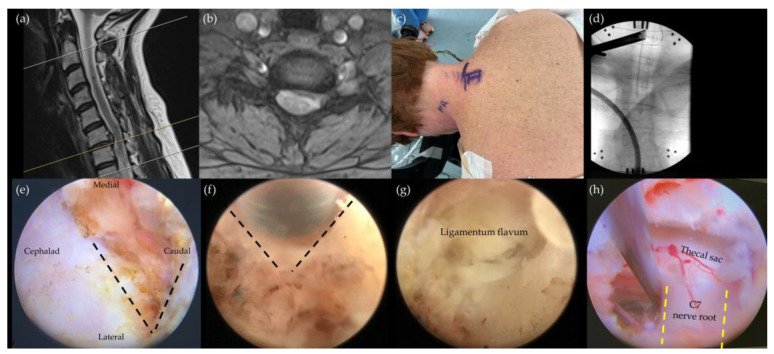
Posterior cervical endoscopic foraminotomy and discectomy for the left C6/7 herniated disc. (**a**,**b**) A soft herniated cervical disc at C6/7 causing compression of the left C7 nerve root. (**c**) The skin incision is to be 2 to 3 mm off the midline, aligned with the C6/7 level. (**d**) The endoscope is docked at the junction of the lamina and the C6/7 facet joint. (**e**) Following the clearance of soft tissues, the V point, indicated by the black dotted lines, is revealed. (**f**) Utilizing an endoscopic bur, bone drilling is performed to widen the surgical field around the V point. (**g**) The lateral section of the ligamentum flavum becomes visible after bone drilling. (**h**) The final image demonstrates the decompressed thecal sac and left C7 nerve root (yellow dotted lines) after removing the ligamentum flavum and herniated disc material.

In 2007, Ruetten et al. [28] introduced the posterior full endoscopic foraminotomy, which further minimizes skin incision and soft tissue damage in the posterior neck. They reported a prospective 2-year study of 87 patients, and the procedure has gained popularity for treating cervical radiculopathy since then. The technique involves positioning patients in a prone posture and docking the endoscope at the “V point” formed by the inferior margin of the cephalic lamina and the medial junction of the inferior and superior facet joints. The subsequent foraminotomy involves precise initial drilling of the lamina and then towards the medial half of the facet joint. After removal of the ligamentum flavum, the thecal sac and exiting nerve root can be identified, enabling targeted discectomy based on clinical requirements (Figure 2). This approach is further refined by techniques such as partial resection of the inferior pedicle to enlarge the neural foramen, enhancing the decompression process [42]. Kim et al. observed a notable increase in decompression area and significant clinical improvements in patients undergoing endoscopic partial pediculotomy and partial vertebrotomy [42].

Posterior endoscopic cervical foraminotomy and discectomy have become the most extensively studied and popular cervical endoscopic techniques. Ruetten and colleagues conducted the first prospective, randomized, controlled study comparing full endoscopic posterior cervical foraminotomy with ACDF [43]. Their findings indicated no significant differences in clinical outcomes, revisions, or complication rates between the two groups. Similarly, another study comparing posterior endoscopic cervical foraminotomy with ACDF reported parallel results in VAS scores, NDI, and modified MacNab criteria, alongside significantly lower blood loss and shorter hospital stays in the PECF group [44]. A recent meta-analysis by Guo et al. comparing the two procedures identified 24 studies with 1345 patients [32]. The study corroborated the findings, showing no statistically significant differences in patients’ effectiveness rate (ACDF: 94.3% vs. PECF: 93.3%), total complication rate (ACDF: 7.1% vs. PECF: 4.7%), and reoperation rate (ACDF: 1.8% vs. PECF: 1.1%) between the two techniques. However, the nature of complications differed between the procedures, with issues such as dysphagia and cage subsidence arising in ACDF and nerve root injury and dural injury arising in PECF.

Concerns regarding postoperative cervical instability and kyphosis have been raised in the context of posterior cervical foraminotomy. A long-term study by Jagannathan et al. [45] reported postoperative loss of lordosis in 20% of patients, with factors such as older age at surgery, preoperative cervical lordosis of less than 10 degrees, and the need for subsequent posterior surgery being associated with worsening sagittal alignment. However, evidence in this area remains mixed, as demonstrated by Won et al., who found that posterior endoscopic cervical foraminotomy effectively reduced radicular symptoms and that preexisting loss of lordosis did not adversely affect outcomes [46].

For patients with a pathology that is localized laterally, posterior endoscopic cervical foraminotomy offers a viable treatment alternative with favorable clinical outcomes. This technique avoids the risks of damaging major vessels, the esophagus, or the trachea. Additionally, preserving the motion segment minimizes the risk of adjacent segment degeneration commonly seen in fusion procedures. Furthermore, posterior endoscopic cervical foraminotomy can serve as a salvage surgery for restenosis following ACDF, eliminating the need to address previous implants or deal with scar tissue [47].

### 4.2. Anterior Endoscopic Cervical Discectomy (AECD)

The anterior endoscopic cervical discectomy technique, which was pioneered in the 1990s, marked a significant advancement in treating cervical herniated discs, demonstrating good clinical results [48,49]. This approach entails the insertion of a guide wire through a carefully delineated safe plane between the carotid sheath and the trachea, followed by the sequential introduction of soft tissue dilators and, finally, the endoscope. The transdiscal route enables the precise removal of the herniated nucleus pulposus, thereby alleviating compression on the spinal cord or cervical nerve roots.

In a study by Ahn et al. [27], 36 patients were followed for a period of 28.6 months. They found that 31 out of the 36 patients experienced either excellent or good outcomes. Echoing these results, another study highlighted significant improvements in pain scores and the neck disability index (NDI) following anterior endoscopic cervical discectomy, with a mean follow-up duration of 45.5 months [30]. However, this technique has raised a concern: Disrupting the cervical disc may accelerate disc degeneration. Despite this, studies have shown that while disc height may decrease post-surgery, the overall and focal sagittal alignments remain largely unaffected. Furthermore, no evidence of segmental instability or spontaneous fusion was noted, and the radiographic changes observed did not appear to impact clinical outcomes [27,30]. In patients presenting with localized soft cervical disc herniation who also have preserved disc height without segmental instability or deformity, anterior endoscopic cervical discectomy preserves the motion segment and minimizes the risk of complications typically associated with fusion procedures.

To mitigate the potential for accelerated disc degeneration, the anterior transcorporeal approach was introduced in 2016. This novel method is characterized by performing the discectomy through the vertebral body rather than directly through the disc [29]. This approach is particularly advantageous for addressing migrated herniated discs, which are often challenging to access via the conventional transdiscal discectomy or anterior cervical discectomy and fusion. Recent studies have documented significant improvements in pain and physical function following this procedure [50,51]. Moreover, the bone tunnel created in the vertebral body during surgery has been shown to heal without any instances of fractures [52]. Although this technique requires a considerable amount of fluoroscopy, its ability to effectively treat pathologies not located at the disc level renders it a promising advancement in cervical spine surgery.

The complication rate of the anterior cervical endoscopic approach was found to be comparable to that of ACDF, with dysphagia being the most common complication. Ahn et al. [53] compared outcomes between 51 patients undergoing endoscopic discectomy and 64 patients undergoing ACDF for single-level soft cervical disc herniation. Three patients in the ACDF group and one in the endoscopic group experienced transient swallowing difficulties. In addition to dysphagia, recurrent disc herniation, hematoma, mediastinal effusion, and hoarseness were identified as less common complications, with the complication rate ranging between 3.75% and 18.5% [54]. Ruetten et al. [37] reported reoperation rates of 6.1% for ACDF, compared to 7.4% for the anterior endoscopic discectomy, which included 49 ACDF and 54 endoscopic cases. The reasons for reoperation in the ACDF group were persistent arm pain and implant-related complications. Conversely, in the endoscopic group, two patients underwent reoperation due to persistent arm pain, and another two did so due to recurrent symptoms following a pain-free interval.

### 4.3. Cervical Endoscopic Unilateral Laminotomy for Bilateral Decompression (CE-ULBD)

The endoscopic unilateral laminotomy for bilateral decompression (ULBD) has proven to be highly effective in the lumbar spine [55,56], and it marks a significant progression in the field of endoscopic spinal surgery. Introduced more recently for the cervical spine [31], this procedure begins by positioning the endoscope at the inferior edge of the cephalic lamina. The surgeon then performs an ipsilateral laminotomy using an endoscopic bur and Kerrison rongeur. For contralateral bony decompression, techniques such as undercutting the base of the spinous process and sublamina drilling, referred to as “over-the-top decompression”, are employed. Removing the ligamentum flavum and exposing both edges of the dural sac are critical steps for ensuring adequate decompression.

Selecting the optimal surgical approach for cervical spondylotic myelopathy depends on several factors, including the number of levels involved, the location of the pathology, and the cervical sagittal alignment [57]. Traditional posterior approaches, such as cervical laminectomy and laminoplasty, have been standard treatments. However, these procedures often entail extensive muscle stripping and wide exposure, leading to significant postoperative axial neck pain and the weakening of posterior cervical paraspinal muscles [8,9]. In contrast, endoscopic unilateral laminotomy for bilateral decompression aims to minimize soft tissue damage and enhance postoperative recovery. A study by Carr et al. [31] involving ten patients revealed significant improvements in the Nurick grade and the modified Japanese Orthopedic Association (JOA) score compared to preoperative values. Further comparative studies, such as those by Yuan [58] and Zhao [59], examined the clinical outcomes between anterior cervical decompression and fusion and endoscopic unilateral laminotomy with bilateral decompression for treating cervical spondylotic myelopathy. These studies reported that hospitalization and operative durations were significantly shorter in the endoscopic group than in the ACDF group. The JOA scores showed significant improvement post-operation in both groups and no significant differences in short-term outcomes. With its array of benefits, including reduced trauma, accelerated recovery, and favorable clinical outcomes, cervical endoscopic unilateral laminotomy for bilateral decompression is a viable alternative for the treatment of cervical myelopathy.

## 5. Limitations of Cervical Endoscopic Spine Surgery

Cervical endoscopic spine surgery, despite its numerous advantages, such as reduced soft tissue dissection, shorter hospital stays, and accelerated functional recovery, confronts several challenges that can limit its broader application.

### 5.1. Steep Learning Curve

One of the primary challenges is the steep learning curve associated with the endoscopic technique. The limited visual field offered by endoscopy contrasts sharply with the expansive view available in open surgery, making it difficult to obtain a comprehensive overview of the anatomy and recognize key landmarks that are crucial for the procedure. Surgeons may find themselves disoriented during surgery, potentially prolonging operation time by being unable to target the pathology directly. Further, this disorientation could increase the risk of damaging neural elements or other vital structures. To ensure smooth surgical procedures, tactile feedback and regular fluoroscopy checks, in addition to visual cues, are recommended. The development and adherence to standardized procedural steps and serial checkpoints, supported by a comprehensive training program, may greatly assist surgeons in navigating the learning curve more effectively.

### 5.2. Radiation Exposure

As with most minimally invasive spine surgeries, endoscopic procedures typically involve greater radiation exposure than in traditional open surgeries [60,61]. Fluoroscopy is essential for initial localization and plays a pivotal role in confirming the actual position intraoperatively. To mitigate radiation exposure, it is imperative that all personnel consistently wear proper lead or equivalent protective shielding, maintain a safe distance of 2 to 3 feet from the X-ray beam source, and position themselves opposite the X-ray source whenever possible [61]. Additionally, using specially designed lead radiation protectors and applying pulsed and collimated X-ray beams have been reported to reduce radiation exposure effectively [62,63].

### 5.3. Limitations with Multiple-Level Lesions

The applicability of cervical endoscopic spine surgery is somewhat confined, with the strongest evidence supporting its use in cases involving soft cervical herniated discs and cervical radiculopathy caused by focal compression [37,44,53,64]. Although there have been a few reports of patients undergoing two-level cervical endoscopic unilateral laminotomy for bilateral decompression in cases of cervical spondylotic myelopathy [31,58], the limited visual field and small working channel inherent to the endoscopic approach can lead to less efficient decompression procedures. Consequently, dealing with diffuse cord compression or multiple-level lesions can be challenging when using endoscopic techniques, rendering traditional open surgery a more feasible and realistic option in such scenarios. However, with appropriate patient selection and surgical indications, favorable surgical outcomes are still achievable despite these limitations.

## 6. Facilitating Endoscopic Surgery

### 6.1. Image-Guided Navigation System/Instrument Tracking System

Disorientation during cervical endoscopic spine surgery is not uncommon, with fluoroscopy playing a critical role in redirecting orientation. However, obtaining fluoroscopic anteroposterior and lateral views can be time-consuming and requires a radiologic technician to ensure procedural safety and smoothness, accompanied by significant radiation exposure. Consequently, advancements in intraoperative imaging represent a crucial step in facilitating endoscopic procedures.

Computed tomography (CT)-guided navigation systems have been successfully integrated into endoscopic spine surgery. In procedures such as percutaneous endoscopic lumbar discectomy, image-guided navigation has proven safe and efficient, enhancing the learning curve and reducing radiation exposure [65]. It has also been beneficial in more complex lumbar spine procedures such as interlaminar contralateral endoscopic lumbar foraminotomy (ICELF) and endoscopic transforaminal lumbar interbody fusion with bilateral decompression [66,67]. For the cervical spine, Zhang et al. [68] reported successful outcomes in percutaneous endoscopic cervical discectomy using O-arm-based navigation, with excellent or good outcomes in 38 out of 42 patients without perioperative complications. Given the mobility of the cervical spine and the small size of pathologies, coupled with the difficulty of visualizing the lower cervical spine due to shoulder obstruction in lateral fluoroscope images, image-guided navigation holds promising potential for advancing cervical endoscopic spine surgery.

**Figure 3:**
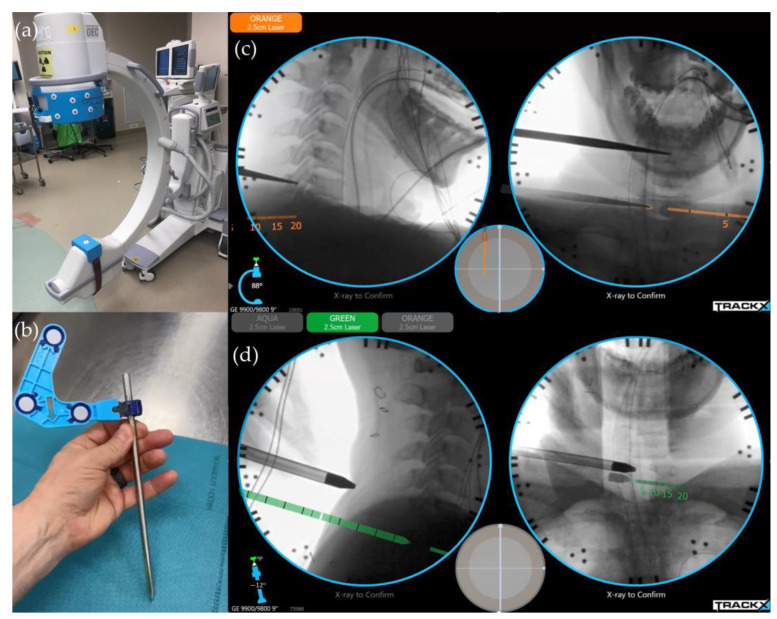
The setup and operation of a real-time surgical instrument tracking system. (**a**) Fiducials are attached to a conventional C-arm, which does not require affixation to the patient or operating table. (**b**) Trackers are secured onto the surgical instruments using quick-connect clamps for efficient attachment. (**c**,**d**) With the instrument tracking system, the surgeon can observe the instrument’s position and trajectory in real time, as the orange and green indicators displayed in both anteroposterior and lateral radiographic views. This feature aids in (**c**) precision skin incision planning and (**d**) ensures accurate dilator placement on the facet joint.

Despite the precision of navigation and reduced radiation exposure for the surgical team, CT-based navigation systems have been found to increase the exposure of the patient to radiation compared to traditional fluoroscopy-based methods [69]. Additionally, any movement of the registered points, including the patient and the trackers, can lead to inaccurate mapping, necessitating repeat scanning [70]. The development of real-time fluoroscopy-based instrument tracking systems—designed to be supplemental to conventional fluoroscopy—eliminates the need for more advanced imaging equipment such as three-dimensional C-arms or intra-operative CT. Studies have demonstrated that these systems reduce radiation exposure and operative time compared to conventional fluoroscopy [71], and they achieve a high degree of instrumentation accuracy that is similar to that of robotic navigation (Figure 3) [72].

### 6.2. Regional Anesthesia for Perioperative Pain Control

Spine surgery ranks among the most painful surgical procedures, and effective control of postoperative pain is crucial for reducing hospital stays and enhancing recovery [73]. Regional anesthesia techniques, such as the erector spinae block (ESP), have been employed in lumbar spine surgeries, significantly reducing postoperative pain [74]. In the cervical spine, cadaver studies have shown that ESP injections at the C6 and C7 levels consistently affect the roots of the brachial plexus and dorsal rami, offering potential analgesia for shoulder and cervical spine surgeries [75]. Although concerns about phrenic nerve paresis exist [76], early case series utilizing ultrasound-guided ESP blocks for cervical and thoracic spine surgeries have demonstrated safety, effective intraoperative and postoperative analgesia, and hemodynamic stability [77]. A double-blind, randomized controlled study in 2023 on using ESP blocks in posterior cervical spine surgery reported significantly lower intraoperative opioid consumption, reduced usage of muscle relaxants, shorter surgical duration, and less intraoperative blood loss in the ESP group. Additionally, this group exhibited better postoperative analgesia and earlier mobilization [78]. Notably, the local anesthetics were administered at the transverse process of T1 in this study, which is considered safer and poses a lower risk of phrenic nerve paresis due to its distance from the vertebral artery [79].

## 7. Future Directions in Cervical Endoscopic Spine Surgery

### 7.1. Minimally Invasive Posterior Cervical Decompression and Fusion

Posterior cervical decompression and fusion are a commonly employed surgical approach for various cervical spine pathologies. Traditional open procedures are often associated with complications such as substantial blood loss, surgical site infections, and pseudoarthrosis [80]. This is attributed to their invasive nature, which impairs local tissue blood flow and prolongs wound exposure. Recently, there has been a growing interest in minimally invasive techniques for cervical spine instrumentation. Coric and Rossi reported their experience with percutaneous posterior cervical pedicle screw instrumentation using navigation guidance in 27 patients. They noted that only three screws required revision [81]. Farah et al. [82] documented their approach of robotically assisted minimally invasive posterior cervical screw fixation, achieving an 85.7% acceptability rate for the screws.

Moreover, there is increasing evidence supporting the clinical efficacy of cervical endoscopic unilateral laminotomy for bilateral decompression in the treatment of cervical stenosis. A novel approach, the percutaneous endoscopic posterior lateral approach, was also recently proposed to address central disc herniation [83]. This technique involves a lateralized skin incision with extended posterior decompression, followed by removing the medial part of the pedicle and employing a smaller diameter endoscope. With this technique, effective decompression of the herniated central disc ventral to the spinal cord is achieved.

Therefore, the integration of minimally invasive cervical spine instrumentation with posterior cervical endoscopic decompression procedures could provide a less invasive alternative to traditional open approaches. This innovative combination holds the promise of expanding the indications for cervical endoscopic surgery and reducing the complications associated with conventional open surgery.

### 7.2. Endoscopic Odontoidectomy and Atlantoaxial Fusion

**Figure 4:**
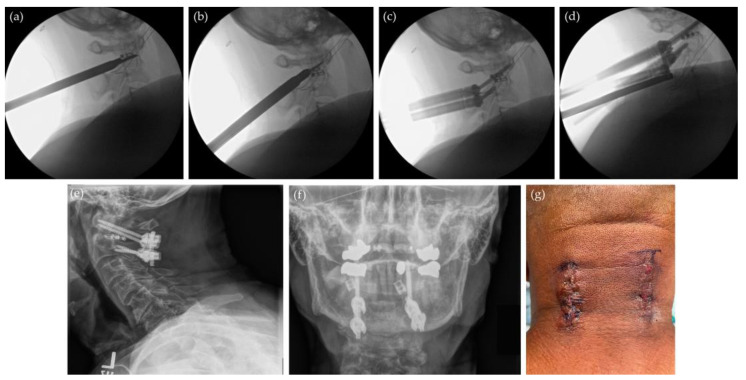
Minimally invasive modification of the Goel–Harms atlantoaxial fusion. Under fluoroscopic guidance and stereotactic navigation, (**a**) intraarticular cages are placed following joint preparation using a distractor. (**b**) The C1 lateral mass is then drilled within the potential space created by the cage placement. Subsequently, (**c**) C1 lateral mass and C2 pars screws with extended tabs are inserted. (**d**) Rods are inserted subfascially to complete the fixation. Postoperative (**e**) lateral and (**f**) anteroposterior radiographs show the implanted instrumentation and the intraarticular cages. (**g**) The procedure is performed via two paramedian skin incisions.

Endoscopic endonasal odontoidectomy (EEO) has emerged as an alternative to the transoral approach for addressing various pathologies at the craniovertebral junction [84]. This technique avoids the need for tongue retraction and palate splitting and lowers the risk of upper airway swelling. Additionally, the endoscope’s proximity to the target area provides a broader surgical view than a surgical microscope through a deep transoral corridor. This enhanced visibility facilitates greater preservation of the C1 arch, thereby minimizing the risk of subsequent atlantoaxial instability and sparing patients from posterior fixation [85]. Should atlantoaxial fixation be necessary, it can be performed using an open approach, a minimally invasive method (Figure 4) [86,87], or an endoscopic-assisted technique [88]. Penner et al. [89] reported on 21 cases treated with endoscopic endonasal odontoidectomy, achieving successful decompression in all patients at the first surgery. Remarkably, in 11 of these patients, posterior instrumentation was unnecessary due to the preservation of the lower portion of the C1 arch, and no significant postoperative spinal instability was observed clinically or radiologically. While two patients experienced intraoperative cerebrospinal fluid (CSF) leaks, there were no postoperative CSF leak complications. Although posterior fixation following odontoidectomy is widely accepted, it has drawbacks, such as the need for a second-stage procedure, extended operative time, and risks associated with posterior spine surgery. To achieve decompression and fixation in a single procedure, Mendes et al. proposed an endoscopic endonasal atlantoaxial transarticular screw fixation technique [90]. This method involves identifying the screw entry point on the anterior aspect of the C1 lateral mass and directing the screw inferiorly and slightly laterally. Biomechanical analysis indicated that this anterior transarticular screw placement is comparable in effectiveness to posterior fixation.

### 7.3. Patient-Specific Surgical Planning

Endoscopic spine surgery is renowned for its ultra-minimally invasive nature, characterized by skin incisions under 2 cm and minimal soft tissue injury. To capitalize on these advantages, precise targeting of pathology is crucial. This precision relies on both meticulous preoperative planning and exact intraoperative execution. High-resolution MRI enables surgeons to delineate the nerve root and measure safe surgical corridors preoperatively [91]. Integrating this detailed preoperative planning with intraoperative navigation systems has shown promising results, as evidenced in a recent study on percutaneous lumbar interbody fusion, which concluded that such integration leads to safer and more successful surgeries [92]. Thus, patient-specific surgical planning is pivotal in ensuring the safety and efficacy of endoscopic procedures.

### 7.4. Application of Augmented Reality (AR) Technology

Augmented reality (AR) represents another frontier in spine surgery, overlaying a virtual environment onto the real world. The most common setup in the operation room is the surgeons’ use of head-mounted displays to visualize intraoperative radiographs or CT-based navigation images superimposed onto the operative field [93,94]. AR technology has been shown to improve the precision of drilling for pedicle screws and reduce the discrepancy in surgeon experience [95]. Studies assessing the accuracy and safety of pedicle screw insertion with AR assistance have reported efficient, reliable, and safe outcomes [96,97]. AR technology has also been applied in various surgical procedures, including minimally invasive lumbar fusion [98], anterior cervical foraminotomy [99], and ACDF for ossification of the posterior longitudinal ligament [100], aiding intraoperative orientation and identification of anatomical landmarks. This technology is particularly beneficial in endoscopic spine surgery, where precise targeting and verification of anatomical landmarks are essential. AR can enhance the efficiency, safety, and learning curve of endoscopic procedures by providing information about the relative positions of bone and neural structures beyond the visual field.

## 8. Conclusions

Cervical endoscopic spine surgery is rapidly evolving and gaining popularity, with novel techniques being continually introduced and scientific evidence increasingly affirming its safety and treatment efficacy. Both anterior and posterior endoscopic approaches to the cervical spine have emerged as alternative treatments for various cervical spine pathologies. The future of cervical endoscopic spine surgery appears promising, with potential enhancements in clinical outcomes and safety. This advancement is driven by the integration of advanced imaging and navigation technologies, the application of regional anesthesia for improved and rapid postoperative recovery, the continuous development of new surgical instruments, and the incorporation of innovative technologies such as augmented reality. With these advancements, cervical endoscopic spine surgery may expand its scope to treat cervical spine pathologies while maintaining its advantages of minimizing tissue damage and facilitating swift recovery.
